# The Correlation Between Visceral Fat Area to Skeletal Muscle Mass Ratio and Multiorgan Insulin Resistance in Chinese Population With Obesity

**DOI:** 10.1155/2024/1297584

**Published:** 2024-10-21

**Authors:** Yanju Zhang, Meiyang Du, Zhouhuiling Li, Xincheng Wang, Mingxin Leng, Yaping Huang, Libin Li, Shi Zhang, Chunjun Li

**Affiliations:** ^1^Tianjin University of Traditional Chinese Medicine, Tianjin 300193, China; ^2^Department of Endocrinology, Health Management Center, Tianjin Union Medical Center, Nankai University Affiliated Hospital, Tianjin, China; ^3^Tianjin Medical University, Tianjin 300070, China; ^4^Department of Endocrinology, Yanshan People's Hospital, Cangzhou 061300, Hebei Province, China

**Keywords:** multiorgan insulin resistance, skeletal muscle mass, visceral fat area

## Abstract

**Aims:** Insulin resistance (IR) is an important risk factor for obesity and cardiometabolic diseases, and our previous findings have demonstrated that visceral fat area to skeletal muscle mass ratio (VSR) is significantly and positively associated with the risk of cardiometabolic diseases. Hence, this study aimed to investigate the relationship between VSR and multiorgan IR, provide a new approach to improve body composition, and set the basis for VSR to increase the incidence of cardiometabolic diseases.

**Materials and Methods:** The study included 398 patients who underwent anthropometric and biochemical measurements, and body composition assessment. Spearman correlation analysis was used to investigate the correlation between VSR and homeostatic model assessment for insulin resistance (HOMA-IR) as well as multiorgan IR, including homeostasis model assessment adiponectin (HOMA-AD), adipose tissue insulin resistance (ADIPO-IR), and hepatic insulin sensitivity (HISI). The new model that incorporated into the present study is made up of easily measured biochemical indicators and is used to predict IR. Logistic regression was used to analyze the odds ratio (OR) of VSR on the risk of multiorgan IR. The predictive value of VSR for HOMA-IR and new model was evaluated using the receiver operating characteristic (ROC) curve.

**Results:** VSR was significantly associated with HOMA-IR, HOMA-AD, ADIPO-IR, 1/HISI, and new model (*p* < 0.001). With the increase of VSR, the OR increased significantly for HOMA-IR and new model (*p* < 0.001). Then, multiorgan IR indicators were quantified, compared to the lowest quartile group, and increased VSR exacerbated the risk of IR in the highest quartile (*p*_trend_ < 0.001). The area under the curve for predicting IR using VSR for HOMA-IR and new model was 0.88 for men, 0.85 for women and 0.73 for men, 0.76 for women, respectively.

**Conclusions:** There was significant correlation between VSR and multiorgan IR, and the risk of multiorgan IR increased with increasing VSR.

**Trial Registration:** Clinical Trial Registry identifier: ChiCTR2100044305.

## 1. Introduction

Obesity is a major public health epidemic globally, and the prevalence of obesity is growing annually. It is predicted that approximately 20% of the global adult population will be threatened by obesity in 2025 [1, 2]. Body mass index (BMI) is the most commonly applied measure of overall obesity in clinical practice; however, it does not reflect where body fat is distributed. It is well known that obesity is a risk factor for insulin resistance (IR) and cardiometabolic diseases, and visceral adipose tissue is a stronger predictor of cardiometabolic risk compared to BMI [3, 4]. Mounting research studies suggest increased visceral fat area (VFA) is an even greater risk factor for IR and cardiometabolic diseases [5, 6], as well as a reduction in skeletal muscle mass (SMM) is closely linked to IR and cardiometabolic diseases [7].

Our previous study found that there was a significant positive association between VFA to SMM ratio (VSR) and cardiometabolic diseases, and with increased VSR, the risk of cardiometabolic diseases also increased [8]. To the best of our knowledge, this is the first epidemiological study reporting the association between VSR and cardiometabolic diseases in a Chinese natural population. But why does VSR affect the risk of cardiometabolic diseases? What are the mechanisms by which VSR leads to the development of cardiometabolic diseases? It prompts the next step of our current research.

IR is one of the main pathological mechanisms of obesity and cardiometabolic diseases, which plays an important role in the development of cardiometabolic diseases [9]. Research clearly showed that IR is closely related to visceral adiposity and patients with IR are more likely to have microvascular complications and cardiometabolic diseases [3, 9]. Therefore, the aim of the present study was to investigate the relationship between VSR and multiorgan IR, which could lay down the pathophysiological mechanisms for further revealing the relationship between VSR and cardiometabolic diseases.

## 2. Methods

### 2.1. Study Population

A cross-sectional survey was conducted in the Health Management Center of Tianjin Union Medical Center. We collected a total of 439 participants included 115 subjects who took an annual health checkup from September 2021 to January 2022 and 324 subjects in the bariatric clinic from January 2021 to June 2022. Participants were excluded if they do not test islet function, or refused to sign the informed consent and provided incomplete information. After excluding 41 individuals, a total of 398 participants were eventually included. Ethical approval was approved by the Medical Ethics Committee of the Tianjin Union Medical Center (No. 2021C06). All participants agreed to participate in the study and signed the informed consent.

### 2.2. Anthropometric and Body Composition Assessment

Following an overnight fasting, the participants were guided to take height and weight measurements with light clothes and barefoot; height and weight were accurate to 0.1 cm and 0.1 kg, respectively; and BMI was calculated based on these data. In the same state, all subjects were tested for body composition analysis. Before starting, the subjects stood in the body composition analyzer (Inbody770, Bio-Space Inc., South Korea) with hands and feet aligned to the four electrodes, separately, then keeping the body upright until the end of the test. Body fat mass (BFM), fat-free mass (FFM), SMM, percent body fat (PBF), and VFA can be detected by this test. All subjects were requests to rest for 10 minutes, and then, the blood pressure was measured using an electronic blood pressure monitor (AC-05C, Ling Qian, China) and the average of the three measurements was the final result.

### 2.3. Biochemical Measurements

Fasting blood samples were taken via venipuncture upon overnight fasting, centrifuged within 12 h, and serum samples were stored in a freezer at −80° until thawed when the samples were needed for analysis. In this study, fasting plasma glucose (FPG), glycosylated hemoglobin (HbA1c), alanine aminotransferase (ALT), aspartate aminotransferase (AST), triglyceride (TG), total cholesterol (TC), low-density lipoprotein cholesterol (LDL-C), high-density lipoprotein cholesterol (HDL-C), and uric acid (UA) were analyzed using an automated biochemical analyzer (TBA120FR, Toshiba, Japan). Fasting insulin levels (FINS) were measured using chemiluminescence immunoassay (Beckman Coulter, China). The detection range was 0.03–300 *μ*U/mL. The intra-assay and interassay coefficients of variation of the enzyme-linked immunosorbent assay (ELISA) kits were < 10% and < 15%. Adiponectin levels was determined using ELISA (Elabscience, Wuhan, China). The minimum detectable concentration was 0.18 ng/mL for adiponectin. The intra-assay and interassay coefficients of variation of the ELISA kits were < 10%.

### 2.4. Definition of Metabolic-Related Diseases

In line with the 2018 Chinese Guidelines for Prevention and Treatment of Hypertension [10], we determined the diagnostic threshold for hypertension as systolic blood pressure (SBP) ≥ 140 mmHg and/or diastolic blood pressure (DBP) ≥ 90 mmHg. According to the 2016 Chinese guidelines for the management of dyslipidemia in adults [11], we defined the hypertensive diagnostic criteria as TG ≥ 2.3 mmol/L or TC ≥ 6.2 mmol/L or HDL-C < 1.0 mmol/L or LDL-C ≥ 4.1 mmol/L. Following the Guideline for the Diagnosis and Management of Hyperuricemia and Gout in China (2019) [12], the diagnostic standard for hyperuricemia was UA ≥ 420 *μ*mol/L. Based on the standards of medical care for type 2 diabetes mellitus (T2DM) in China (2019) [13], hyperglycemia was defined as FPG ≥ 7.0 mmol/L and/or 2-h postprandial blood glucose by oral glucose tolerance test (OGTT) ≥ 11.1 mmol/L.

### 2.5. Calculation and Definition of Multiorgan IR

The formula for calculating homeostatic model assessment for insulin resistance (HOMA-IR) [14] was [FINS (*μ*U/mL) × FPG (mmol/L)]/22.5. According to the Chinese epidemiological surveys [15], the cutoff value for the diagnosis of HOMA-IR was 2.69. The formula for calculating homeostasis model assessment adiponectin (HOMA-AD) [16] was [FPG (mmol/L) × FINS (*μ*U/mL)]/adiponectin (*μ*g/mL). The formula for calculating adipose tissue insulin resistance (ADIPO-IR) [17] was free fatty acid (FFA) (mmol/L) × FINS (*μ*U/mL). The formula for calculating hepatic insulin sensitivity (HISI) was *k*/(FPG × FINS), and the *k* was 22.5 × 18 [18]. In this article, we used the inverse of HISI to represent hepatic IR, which will facilitate our subsequent analysis. Research showed visceral adiposity is crucial in the evolution of IR, and vicarious indicators that reflected visceral obesity may have a higher predictive value for IR. Therefore, our team had established and validated a new IR prediction model [19] that includes visceral obesity indicators and confirmed its predictive value by comparing it with other vicarious indicators. The formula used to calculate the new model was (0.0293 × age + 1.4892 × Ln VFA + 0.4966 × Ln TG + 2.784 × Ln FPG + 0.6906 × Ln ALT)/2, and the cutoff value for the new model was 8.18.

### 2.6. Statistical Analysis

All data were verified accurately and then grouped by gender and imported into SPSS26.0 statistical software for data analysis. Continuous variables were tested for normality, and the Kolmogorov–Smirnov test or Shapiro–Wilk test was chosen depending on whether the sample size was greater than 100. Continuous numeric variables with a normal distribution were expressed as mean ± standard deviation, while non-normally distributed variables were expressed as median and interquartile range P50 (P25, P75). Categorical variables were expressed as frequencies and percentages [*n* (%)]. Analysis of variance between groups was performed using the unpaired *T*-test for continuous variables that obeyed a normal distribution, the Mann–Whitney *U* test for continuous variables that did not obey a normal distribution or obeyed a normal distribution with uneven variance, and the chi-square test for categorical variables. To investigate the correlation between VSR and multiorgan IR, the Spearman correlation analysis was performed. In order to evaluate the prevalence risk of multiorgan IR, a multiple logistic regression analysis approach was applied, with multiorgan IR index and the new predictive model for resistance as dependent variables and VSR as an independent variable, and receiver operating characteristic (ROC) curves were used to estimate the diagnostic value of VSR for multiorgan IR risk.

## 3. Results

### 3.1. Clinical Characteristics of the Study Population

A total of 398 participants were recruited in this study. All the subjects were divided into two groups based on gender, and each group was further divided into two subgroups. Based on previous research by our team, the risk of cardiometabolic diseases was relatively flat until VSR reached 3.078 cm^2^/kg in men and 4.750 cm^2^/kg in women and started to increase rapidly afterward. Therefore, we chose these two values as the basis for the grouping criteria. The clinical characteristics of the study are presented in Table 1. Regardless of gender, subjects in G2 had higher BMI, PBF, VFA, SBP, DBP, FPG, FIN, HbA1c, UA, ALT, AST, TC, TG, and LDL-C. Moreover, the overall prevalence of hypertension, hyperlipidemia, hyperuricemia and hyperglycemia was higher in G2 than in G1.

### 3.2. Association Between VSR and Multiorgan IR

**Figure 1:**
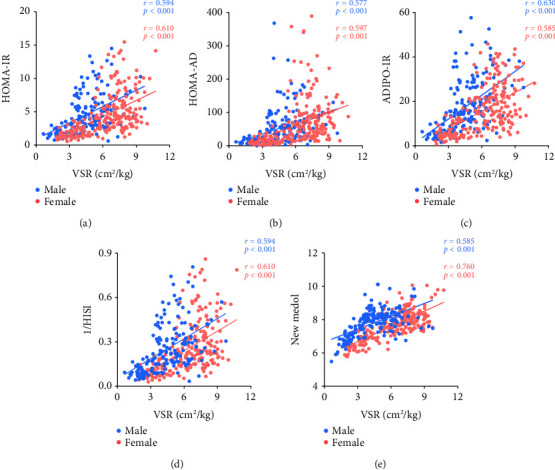
Correlation between multiorgan insulin resistance and VSR in male and female subjects. ADIPO-IR, adipose tissue insulin resistance; HISI, hepatic insulin sensitivity; HOMA-AD, homeostasis model assessment adiponectin; HOMA-IR, homeostatic model assessment for insulin resistance; VSR, visceral fat area to skeletal muscle mass ratio.

**Figure 2:**
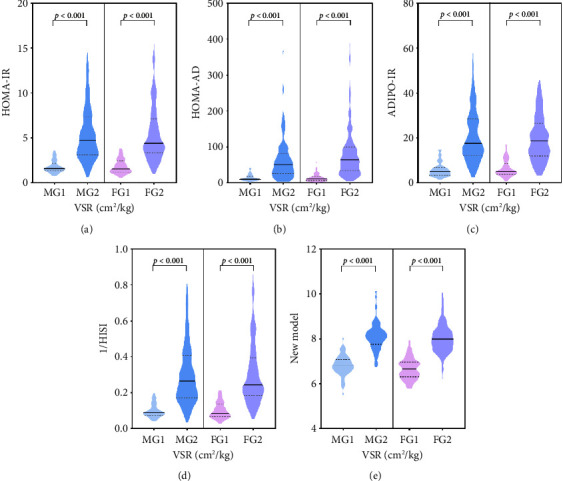
Multiorgan insulin resistance in different groups. Male: MG1: VSR ≤ 3.078 (cm^2^/kg), MG2: VSR > 3.078 (cm^2^/kg); female: FG1: VSR ≤ 4.750 (cm^2^/kg), FG2: VSR > 4.750 (cm^2^/kg). ADIPO-IR, adipose tissue insulin resistance; HISI, hepatic insulin sensitivity; HOMA-AD, homeostasis model assessment adiponectin; HOMA-IR: homeostatic model assessment for insulin resistance, VSR, visceral fat area to skeletal muscle mass ratio.

In order to evaluate the association between VSR and multiorgan IR, the correlation between VSR and multiorgan IR by Spearman analysis is presented in Figure 1. We could observe a moderate strength correlation between VSR and multiorgan IR. Multiorgan IR in different groups of male and female is demonstrated in Figure 2. There were remarkable differences of VSR and multiorgan IR both in male and female (*p* < 0.001), and we could see that multiorgan IR in G1 is significantly higher than in G2 (*p* < 0.001).

Binary logistic regression analysis was performed to further analyze the associations between VSR and HOMA-IR, new model in Table 2. In Model 1, the odds ratio (OR) with 95% confidence interval (CI) in male and female were 3.33 (2.31, 4.80) and 2.21 (1.80, 2.70) for HOMA-IR and 1.53 (1.26, 1.87) and 1.80 (1.46, 2.22) for new model, respectively (*p* < 0.05). Simultaneously, this association is also present in the adjusted Model 2 and Model 3. We can also find that the OR of VSR for HOMA-IR in male is higher than in female.

**Figure 3:**
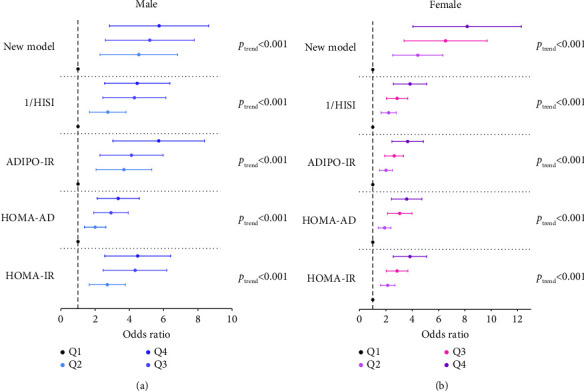
OR with 95% CI for VSR according to multiorgan insulin resistance quartile. ADIPO-IR, adipose tissue insulin resistance; CI, confidence interval; OR, odds ratio; HISI, hepatic insulin sensitivity; HOMA-AD, homeostasis model assessment adiponectin; HOMA-IR, homeostatic model assessment for insulin resistance; VSR, visceral fat area to skeletal muscle mass ratio.

In order to further clearly express the degree of influence of VSR on aggravating the risk of multiorgan IR, we analyzed the multiorgan IR indicators in four equal parts according to gender. In Figure 3, the results showed that all multiorgan IR indicators were quantified; the OR for the highest quartile HOMA-IR was 4.21 (95% CI: 2.71–6.54) and 3.68 (2.63–5.16) for men and women, respectively; the OR for the highest quartile HOMA-AD was 3.20 (2.20–4.64) and 3.46 (2.51–4.79); the OR for the highest quartile ADIPO-IR was 5.30 (3.27–8.58) and 3.52 (2.53–4.91); the OR for the highest quartile 1/HISI was 4.19 (2.70–6.49) and 3.70 (2.64–5.16); and the OR for the highest quartile new model was 5.25 (3.11–8.84) and 7.48 (4.44–12.59).

### 3.3. ROC Curve of VSR for Prediction of HOMA-IR and New Model

**Figure 4:**
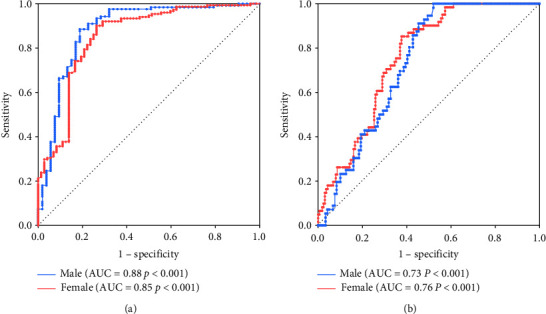
(a) ROC curve analysis of VSR prediction of HOMA-IR in different genders. (b) ROC curve analysis of VSR prediction of new model in different genders. ADIPO-IR, adipose tissue insulin resistance; HISI, hepatic insulin sensitivity; HOMA-AD, homeostasis model assessment adiponectin; HOMA-IR, homeostatic model assessment for insulin resistance; ROC, receiver operating characteristic; VSR, visceral fat area to skeletal muscle mass ratio.

ROC curve analysis was used to describe the predictive value of VSR for HOMA-IR and new model in Figure 4. The area under the ROC curve for HOMA-IR and new model in male and female subjects was 0.88 and 0.85, and 0.73 and 0.76, respectively. The optimal cutoff value of HOMA-IR in male subjects was 3.53 with a sensitivity of 89% and a specificity of 81%; in female subjects, it was 4.83 with a sensitivity of 92% and a specificity of 71%. Moreover, the cutoff value, sensitivity, and specificity for new model were 3.53%, 100%, 48% and 5.36%, 99%, 43% in male and female.

## 4. Conclusion

In the present study, we found a significant positive correlation between VSR and all indicators of multiorgan IR, while all indicators of IR were significantly higher in the high-VSR group than in the low-VSR group. Similarly, the risk of multiorgan IR increased progressively with increasing VSR, and the results still held after calibration for confounding factors. Neither of these results differed by gender. In addition to that, we performed a ROC analysis and found that VSR had a strong predictive effect on IR.

The swift progression of China's economy and the adoption of westernized lifestyles have resulted in a rise in overweight and obesity prevalence over time. This phenomenon presents a significant obstacle to public health in both developing and developed nations, as it is linked to a multitude of illnesses and heightened all-cause mortality [4]. Numerous epidemiological studies have established that obesity constitutes a primary risk factor for cardiometabolic diseases [20]. Nevertheless, not all individuals who are obese are vulnerable to these conditions, as some “metabolically healthy obese” individuals exhibit normal insulin sensitivity despite being overweight, but unhealthy patients with obesity are mainly characterized by excessive visceral fat content. Therefore, people with the same BMI who have different body composition are quite different risks in the progression of cardiometabolic diseases [21].

In nearly a decade of research, we have observed that VFA is a more reflective indicator of physical health and cardiometabolic diseases as opposed to BMI or waist-to-hip ratio [22, 23]. Meanwhile, the accumulation of visceral fat was the major contributor to IR [24]. Increased visceral adipose tissue promoted the release of numerous inflammatory cytokines, such as interleukin-6 (IL-6), tumor necrosis factor-alpha (TNF-*α*), and interleukin-1beta (IL-1*β*), leading to a state of systemic hypoinflammation [25]. The inflammatory state of adipose tissue reduced the production and secretion of adiponectin, which led to a reduction in insulin sensitivity. IR hampered the uptake and utilization of glucose, and excess insulin can be secreted, enhancing the catabolism of peripheral adipose tissue and releasing excess FFA into the blood, promoting the synthesis of adipose tissue in the liver, leading to visceral fat accumulation. Visceral fat accumulation can further block insulin signaling pathways, thereby increasing IR. IR was the underlying cause of the metabolic syndrome (MS) in patients with obesity and an influential pathogenic mechanism for cardiometabolic diseases [26]. Skeletal muscle is an important endocrine organ, which involved in the key sites of insulin-induced glucose metabolism, maintaining normal glucose homeostasis environment. The decrease of SMM can lead to the disruption of glucose metabolism in internal environment, resulting in IR [27, 28]. At the same time, IR contributes to reduced muscle protein synthesis and accelerated muscle protein catabolism, which promotes skeletal muscle loss and may eventually lead to type 2 diabetic sarcopenia [29]. Skeletal muscle is the target of obesity-induced inflammation, while the obesity-induced inflammation and IR can also cause the release of specific skeletal muscle cytokines, such as IL-6, irisin, myostatin, and muscle growth inhibitor [30, 31]. The release of these cytokines can increase or decrease the risk of obesity, inflammation, and IR [32, 33]. Overall, skeletal muscle loss is strongly associated with IR.

The distinctive feature of IR is its tissue specificity, which can involve the liver, muscle, and adipose tissue, and IR in different tissues causes varying degree effects on the body. The liver is the first organ where insulin reaches after being secreted from the pancreas and it regulates both storage and disposal of glucose in response to the requirements of the body for insulin. As the most sensitive organ to IR, hepatic IR is also the main cause of IR in other peripheral organs and tissues. Skeletal muscle and adipose tissue are two primary target organs for glucose disposal. The infiltration of lipids into myocytes produces massive amounts of reactive oxygen species, which in turn induces the release of calcium ions, cytochrome C, and apoptosis-inducing factors. Intramuscular lipids also trigger mitochondrial dysfunction, as evidenced by impaired *β*-oxidation capacity, and the numerous damaged mitochondria are removed by autophagy in the body, resulting in a decline in both mitochondrial density and function, impairing the oxidative capacity of skeletal muscle cells, and further leading to lipid infiltration, ectopic lipid deposition, and the progression IR [34]. Hypertrophic adipose tissue has the capacity to release excess FFA and proinflammatory cytokines; once excess FFA and dietary lipids enter the cells of nonadipose tissue such as liver, muscle, and pancreas as ectopic fat deposits, there will be stepwise development of lipotoxicity [35]. The development of lipotoxicity sets the stage for IR and chronic low-grade systemic inflammation [36].

HOMA-IR is a reliable alternate predictor of IR in addition to hyperinsulinemic–euglycemic clamp technique. We demonstrated that VSR is an independent risk factor for IR and also has a high predictive ability for IR, which clearly indicates a strong relationship between VSR and IR. Insulin has different functions in organ system; in the liver, it reduces hepatic glucose production; and in adipose tissue, it inhibits lipolysis. While hepatic IR is considered an early event in peripheral IR and usually precedes the onset of obesity and other metabolism-related diseases, hepatic IR has the potential to contribute to cardiovascular disease and lead to patient morbidity and mortality through a vicious circle [37]. ADIPO-IR, as a unique predictor of IR in adipose tissue, may reflect the antilipolytic effect of insulin in adipose tissue [14], as well as suggesting progression of aortic valve calcification and adverse cardiovascular features [38]. Findings suggest that HOMA-AD is more accurate at assessing IR than HOMA-IR in individuals with overweight nondiabetes [39]. Increased VSR in the highest quartile exacerbated the risk of 1/HISI, ADIPO-IR, and HOMA-AD, compared to the lowest quartile group. The results reveal a close relationship between them. Based on our previous study, we demonstrated the validity of the new model while the model is based on easily available and inexpensive parameters. It is well known that visceral adiposity plays a key role in the development of IR. Therefore, using surrogate indicators that include visceral fat may have a higher predictive value than indicators that use only biochemical measures and this conjecture has been confirmed in the previous study [19]. Then, we conducted an analysis using the new model with the VSR, resulting in similar results as HOMA-IR, with the VSR being an independent risk factor for the new model, while also having the same strength of predictive power for the new model resistance.

IR is widely acknowledged as a significant risk factor for vascular stiffness and the development of adverse cardiometabolic diseases [40, 41]. In a state of normal physiological functioning, insulin stimulates the production of nitric oxide by vascular endothelial cells, thereby promoting vasodilation and increasing blood flow [42]. However, in a state of IR, the sensitivity of insulin to vascular action is diminished and may even exacerbate vessel constriction by upregulating the production of vasoconstrictive factors such as endothelin, leading to pathological vascular sclerosis [42, 43]. An abnormal increase in vascular stiffness has been demonstrated to be an independent predictor of cardiometabolic diseases. In the inflammatory state of the body, the increased plasma concentrations of proinflammatory cytokines, IL-1*β*, IL-6, and TNF-*α*, and C-reactive protein (CRP) reflected a stronger propensity for cardiometabolic diseases [44, 45]. Consequently, IR is a risk factor for cardiometabolic diseases and increases the prevalence of cardiometabolic diseases [46].

In general, the results of the current study revealed a positive correlation between VSR and multiorgan IR, with the effect of VSR on the risk of IR increasing progressively as IR worsens. At the same time, VSR is a strong predictor of IR index, namely, HOMA-IR and new model. As a new indicator, VSR could provide new ideas for weight loss in individuals with obesity. We consider that VSR as a key target to help promote health in large populations, through accessible and scalable dietary-exercise modality interventions to improve body composition, reduce visceral fat, increase SMM, mitigate, or even reverse the progression of cardiometabolic diseases, and in the long run, the benefits are also enormous for the minimization of CVD event mortality. This is also a direction that our team will continue to work on in the future.

Drawing on the previous team's research, this study further explores the mechanisms behind VSR in depth. We summarize the intense relationship between VSR and IR, which is of great significance in guiding the improvement of body composition and promoting physical health. The drawbacks of the research are that it is only a cross-sectional study, which cannot judge the causal relationship between VSR and IR, as well as the small sample size of the present study. With the hope that future experiments based on the present study can explore how improvements in VSR can improve IR in population with obesity.

## Figures and Tables

**Table 1:** Clinical characteristics of the study population according to gender and VSR status^b^.

Characteristics	Male		*p*	Female		*p*
	MG1	MG2		FG1	FG2	
*N* (%)	45 (25.71)	130 (74.29)		61 (27.35)	162 (72.65)	
Age, years	33.87 ± 8.17	35.29 ± 13.67	0.407	32.97 ± 7.28	34.33 ± 11.64	0.299
Height, cm	175.36 ± 5.75	173.47 ± 7.77	0.137	164.84 ± 5.38	162.80 ± 5.85	**0.018**
Weight, kg	71.24 ± 10.37	104.21 ± 21.89	**< 0.001**	57.91 ± 8.55	89.40 ± 17.56	**< 0.001**
BMI, kg/m^2^	23.35 ± 3.10	34.48 ± 6.11	**< 0.001**	21.35 ± 2.64	33.66 ± 6.08	**< 0.001**
BFM, kg	14.90 (13.80, 16.60)	38.00 (30.50, 50.30)	**< 0.001**	16.30 (14.00, 19.30)	39.20 (32.90, 46.30)	**< 0.001**
FFM, kg	56.08 ± 6.26	63.00 ± 10.44	**< 0.001**	41.60 ± 4.83	48.58 ± 7.03	**< 0.001**
SMM, kg	31.49 ± 3.75	35.35 ± 6.35	**< 0.001**	22.44 ± 2.89	26.72 ± 4.22	**< 0.001**
PBF, %	20.86 ± 3.92	38.56 ± 6.13	**< 0.001**	28.21 ± 4.53	44.80 ± 4.89	**< 0.001**
VFA, cm^2^	65.89 ± 20.65	182.19 ± 53.76	**< 0.001**	73.20 ± 21.18	195.28 ± 41.88	**< 0.001**
SBP, mmHg	121.42 ± 11.38	139.64 ± 16.93	**< 0.001**	115.03 ± 11.15	133.02 ± 19.90	**< 0.001**
DBP, mmHg	73.93 ± 10.02	84.32 ± 13.97	**< 0.001**	68.75 ± 7.40	80.41 ± 13.06	**< 0.001**
FPG, mmol/L	4.83 ± 0.44	5.50 ± 1.86	**< 0.001**	4.65 ± 0.55	5.49 ± 1.54	**< 0.001**
FIN, *μ*U/mL	7.45 (6.34, 10.04)	18.92 (14.64, 26.94)	**< 0.001**	7.59 (6.17, 10.23)	19.49 (15.51, 27.96)	**< 0.001**
HbA1c, %	5.30 (5.20, 5.50)	5.75 (5.40, 6.10)	**< 0.001**	5.40 (5.30, 5.60)	5.70 (5.40, 6.10)	**< 0.001**
UA, *μ*mol/L	356.44 ± 87.98	439.82 ± 126.36	**< 0.001**	245.33 ± 53.10	370.94 ± 99.91	**< 0.001**
ALT, mmol/L	20.00 (15.00, 25.00)	37.90 (26.90, 68.10)	**< 0.001**	13.00 (11.00, 16.00)	26.40 (17.50, 46.60)	**< 0.001**
AST, mmol/L	20.00 (17.00, 23.00)	26.25 (21.40, 38.00)	**< 0.001**	16.00 (14.00, 18.00)	24.10 (18.00, 35.90)	**< 0.001**
TC, mmol/L	4.43 (3.82, 4.89)	4.75 (4.27, 5.69)	**0.009**	4.50 (4.15, 5.17)	4.94 (4.28, 5.67)	**0.022**
TG, mmol/L	0.91 (0.73, 1.07)	1.58 (1.22, 2.47)	**< 0.001**	0.80 (0.66, 1.16)	1.51 (1.16, 2.21)	**< 0.001**
HDL-C, mmol/L	1.45 (1.26, 1.61)	1.15 (1.02, 1.28)	**< 0.001**	1.66 (1.41, 1.91)	1.26 (1.05, 1.56)	**< 0.001**
LDL-C, mmol/L	2.58 (2.12, 3.21)	3.07 (2.63, 3.66)	**0.001**	2.68 (2.37, 3.05)	3.04 (2.49, 3.61)	**0.003**
Hypertension, *n* (%)	3 (6.70)	66 (50.80)	**< 0.001**	1 (1.60)	58 (35.80)	**< 0.001**
Hyperlipidemia, *n* (%)	5 (11.10)	60 (46.20)	**< 0.001**	5 (8.20)	59 (36.40)	**< 0.001**
Hyperuricemia, *n* (%)	6 (13.30)	72 (55.40)	**< 0.001**	0 (0.00)	42 (25.90)	**< 0.001**
Hyperglycemia, *n* (%)	0 (0.00)	66 (50.80)	**< 0.001**	2 (3.30)	91 (56.20)	**< 0.001**
HOMA-IR	1.60 (1.34, 2.16)	4.76 (3.12, 7.32)	**< 0.001**	1.54 (1.19, 2.45)	4.38 (3.34, 7.11)	**< 0.001**
HOMA-AD	10.50 (8.24, 18.40)	51.09 (26.71, 82.41)	**< 0.001**	11.44 (6.71, 17.97)	64.46 (34.72, 99.62)	**< 0.001**
ADIPO-IR	5.14 (3.33, 6.86)	17.55 (12.22, 28.46)	**< 0.001**	5.14 (3.82, 8.64)	18.69 (12.00, 26.47)	**< 0.001**
1/HISI	0.09 (0.07, 0.12)	0.26 (0.17, 0.40)	**< 0.001**	0.09 (0.07, 0.14)	0.24 (0.19, 0.40)	**< 0.001**
New model	6.81 (6.53, 7.10)	8.10 (7.76, 8.37)	**< 0.001**	6.66 (6.30, 6.97)	7.99 (7.54, 8.37)	**< 0.001**

*Note:* (a) Continuous data were presented as mean ± standard deviation or median (interquartile range). Categorical variables were expressed as frequency or percentage. (b) Male: MG1: VSR ≤ 3.078 (cm^2^/kg), MG2: VSR > 3.078 (cm^2^/kg); female: FG1: VSR ≤ 4.750 (cm^2^/kg), FG2: VSR > 4.750 (cm^2^/kg). The bold values represent that all *p* values are meaningful, and b explains the cut-off values of VSR for different genders.

Abbreviations: ADIPO-IR, adipose tissue insulin resistance; ALT, alanine transaminase; AST, aspartate transaminase; BFM, body fat mass; BMI, body mass index; DBP, diastolic blood pressure; FFM, fat-free mass; FIN, fasting insulin; FPG, fasting plasma glucose; HbA1c, glycated hemoglobin; HDL-C, high-density lipoprotein cholesterol; HISI, hepatic insulin sensitivity; HOMA-AD, homeostasis model assessment adiponectin; HOMA-IR, homeostatic model assessment for insulin resistance; LDL-C, low-density lipoprotein cholesterol; PBF, percent body fat; SBP, systolic blood pressure; SMM, skeletal muscle mass; SUA, serum uric acid; TC, total cholesterol; TG, triglycerides; VFA, visceral fat area; VSR: visceral fat area to skeletal muscle mass ratio.

**Table 2:** Logistic regression analysis for VSR according to HOMA-IR and new model.

Variables	Male			Female		
	Model 1	Model 2	Model 3	Model 1	Model 2	Model 3
HOMA-IR	3.33 (2.31, 4.80)⁣^∗∗∗^	2.68 (1.80, 3.98)⁣^∗∗∗^	2.16 (1.45, 3.21)⁣^∗∗∗^	2.21 (1.80, 2.70)⁣^∗∗∗^	2.04 (1.63, 2.53)⁣^∗∗∗^	1.73 (1.33, 2.24)⁣^∗∗∗^
New model	1.53 (1.26, 1.87)⁣^∗∗∗^	1.54 (1.14, 2.07)⁣^∗∗^	1.81 (1.15, 2.87)⁣^∗^	1.80 (1.46, 2.22)⁣^∗∗∗^	1.80 (1.37, 2.36)⁣^∗∗∗^	1.57 (1.06, 2.32)⁣^∗^

*Note:* Model 1: not adjusted. Model 2: adjusted for age, SBP, and HbA1c. Model 3: adjusted for UA, ALT, TC, and HDL-L based on Model 2.

Abbreviation: HOMA-IR, homeostasis model assessment for insulin resistance.

⁣^∗^*P* < 0.05; ⁣^∗∗^*P* < 0.01; ⁣^∗∗∗^*P* < 0.001.

## Data Availability

The data used to support the findings of this study are available from the corresponding author upon request.

## References

[1] World Health Organization (2021). *WHO Discussion Paper: Draft Recommendations for the Prevention and Management of Obesity Over the Life Course, Including Potential Targets*.

[2] Flegal K. M. (2021). BMI and Obesity Trends in Chinese National Survey Data. *The Lancet*.

[3] Hulkoti V., Acharya S., Shukla S. (2022). Visceral Adiposity Index in Type 2 Diabetes Mellitus (DM) and Its Correlation With Microvascular Complications. *Cureus*.

[4] Pan X. F., Wang L., Pan A. (2021). Epidemiology and Determinants of Obesity in China. *Lancet Diabetes & Endocrinology*.

[5] He M., Wang J., Liang Q. (2022). Time-Restricted Eating With or Without Low-Carbohydrate Diet Reduces Visceral Fat and Improves Metabolic Syndrome: A Randomized Trial. *Cell Reports Medicine*.

[6] Valenzuela P. L., Carrera-Bastos P., Castillo-García A., Lieberman D. E., Santos-Lozano A., Lucia A. (2023). Obesity and the Risk of Cardiometabolic Diseases. *Nature Reviews Cardiology*.

[7] Heo J. E., Shim J. S., Lee H., Kim H. C. (2020). Association Between the Thigh Muscle and Insulin Resistance According to Body Mass Index in Middle-Aged Korean Adults. *Journal of Diabetes & Metabolism*.

[8] Zhang S., Huang Y., Li J. (2023). Increased Visceral Fat Area to Skeletal Muscle Mass Ratio is Positively Associated With the Risk of Cardiometabolic Diseases in a Chinese Natural Population: A Cross-Sectional Study. *Diabetes*.

[9] Zhao X., An X., Yang C., Sun W., Ji H., Lian F. (2023). The Crucial Role and Mechanism of Insulin Resistance in Metabolic Disease. *Frontiers in Endocrinology*.

[10] Joint Committee for Guideline Revision (2019). 2018 Chinese Guidelines for Prevention and Treatment of Hypertension-A Report of the Revision Committee of Chinese Guidelines for Prevention and Treatment of Hypertension. *Journal of Geriatric Cardiology*.

[11] Joint Committee for Guideline Revision (2018). 2016 Chinese Guidelines for the Management of Dyslipidemia in Adults. *Journal of Geriatric Cardiology*.

[12] Association C. S. o E. C. M. (2020). Guideline for the Diagnosis and Management of Hyperuricemia and Gout in China (2019). *Chinese Journal of Endocrinology and Metabolism*.

[13] Jia W., Weng J., Zhu D. (2019). Standards of Medical Care for Type 2 Diabetes in China 2019. *Diabetes*.

[14] Zhang K., Pan H., Wang L., Yang H., Zhu H., Gong F. (2021). Adipose Tissue Insulin Resistance is Closely Associated With Metabolic Syndrome in Northern Chinese Populations. *Diabetes, Metabolic Syndrome and Obesity: Targets and Therapy*.

[15] Xiao-yan X., Wwn-ying Y., Zhao-jun Y. (2004). The Diagnostic Significance of Homeostasis Model Assessment of Insulin Resistance in Metabolic Syndrome Among Subjects With Different Glucose Tolerance. *Chinese Journal of Diabetes*.

[16] Matsuhisa M., Yamasaki Y., Emoto M. (2007). A Novel Index of Insulin Resistance Determined From the Homeostasis Model Assessment Index and Adiponectin Levels in Japanese Subjects. *Diabetes Research and Clinical Practice*.

[17] Gastaldelli A., Gaggini M., DeFronzo R. A. (2017). Role of Adipose Tissue Insulin Resistance in the Natural History of Type 2 Diabetes: Results From the San Antonio Metabolism Study. *Diabetes*.

[18] Matsuda M., DeFronzo R. A. (1999). Insulin Sensitivity Indices Obtained from Oral Glucose Tolerance Testing: Comparison With the Euglycemic Insulin Clamp. *Diabetes Care*.

[19] Zhang S., Wang X. C., Li J. (2022). Establishment and Validation of a New Predictive Model for Insulin Resistance Based on 2 Chinese Cohorts: A Cross-Sectional Study. *International Journal of Endocrinology*.

[20] Forouzanfar M. H., Reitsma M. B., Sur P. (2017). Health Effects of Overweight and Obesity in 195 Countries over 25 Years. *New England Journal of Medicine*.

[21] Neeland I. J., Ross R., Després J. P. (2019). Visceral and Ectopic Fat, Atherosclerosis, and Cardiometabolic Disease: A Position Statement. *Lancet Diabetes & Endocrinology*.

[22] Kim S. H., Kang H. W., Jeong J. B. (2021). Association of Obesity, Visceral Adiposity, and Sarcopenia With an Increased Risk of Metabolic Syndrome: A Retrospective Study. *PLoS One*.

[23] Franek E., Pais P., Basile J. (2023). General Versus Central Adiposity as Risk Factors for Cardiovascular-Related Outcomes in a High-Risk Population with Type 2 Diabetes: A Post Hoc Analysis of the REWIND Trial. *Cardiovascular Diabetology*.

[24] Stefan N. (2020). Causes, Consequences, and Treatment of Metabolically Unhealthy Fat Distribution. *Lancet Diabetes & Endocrinology*.

[25] Wu H., Ballantyne C. M. (2020). Metabolic Inflammation and Insulin Resistance in Obesity. *Circulation Research*.

[26] Gallagher E. J., Leroith D., Karnieli E. (2010). Insulin Resistance in Obesity as the Underlying Cause for the Metabolic Syndrome. *Mount Sinai Journal of Medicine: A Journal of Translational and Personalized Medicine*.

[27] Seko T., Akasaka H., Koyama M. (2019). Lower Limb Muscle Mass is Associated With Insulin Resistance More Than Lower Limb Muscle Strength in Non-Diabetic Older Adults. *Geriatrics and Gerontology International*.

[28] Nishikawa H., Asai A., Fukunishi S., Nishiguchi S., Higuchi K. (2021). Metabolic Syndrome and Sarcopenia. *Nutrients*.

[29] Liu Z. J., Zhu C. F. (2023). Causal Relationship Between Insulin Resistance and Sarcopenia. *Diabetology & Metabolic Syndrome*.

[30] Gomarasca M., Banfi G., Lombardi G. (2020). Myokines: The Endocrine Coupling of Skeletal Muscle and Bone. *Advances in Clinical Chemistry*.

[31] Remuzgo-Martínez S., Rueda-Gotor J., Pulito-Cueto V. (2022). Irisin as a Novel Biomarker of Subclinical Atherosclerosis, Cardiovascular Risk and Severe Disease in Axial Spondyloarthritis. *Frontiers in Immunology*.

[32] Merz K. E., Thurmond D. C. (2020). Role of Skeletal Muscle in Insulin Resistance and Glucose Uptake. *Comprehensive Physiology*.

[33] Kim H. K., Kim C. H. (2021). Quality Matters as Much as Quantity of Skeletal Muscle: Clinical Implications of Myosteatosis in Cardiometabolic Health. *Endocrinology and Metabolism (Seoul)*.

[34] Rubio-Ruiz M. E., Guarner-Lans V., Pérez-Torres I., Soto M. E. (2019). Mechanisms Underlying Metabolic Syndrome-Related Sarcopenia and Possible Therapeutic Measures. *International Journal of Molecular Sciences*.

[35] Ahmed B., Sultana R., Greene M. W. (2021). Adipose Tissue and Insulin Resistance in Obese. *Biomedicine & Pharmacotherapy*.

[36] Milano W., Carizzone F., Foia M. (2022). Obesity and Its Multiple Clinical Implications Between Inflammatory States and Gut Microbiotic Alterations. *Diseases*.

[37] Meshkani R., Adeli K. (2009). Hepatic Insulin Resistance, Metabolic Syndrome and Cardiovascular Disease. *Clinical Biochemistry*.

[38] Jorge-Galarza E., Posadas-Romero C., Torres-Tamayo M. (2016). Insulin Resistance in Adipose Tissue but Not in Liver is Associated With Aortic Valve Calcification. *Disease Markers*.

[39] Branda J. I. F., de Almeida-Pititto B., Bensenor I., Lotufo P. A., Ferreira S. R. G. (2022). Low Birth Weight, *β*-Cell Function and Insulin Resistance in Adults: The Brazilian Longitudinal Study of Adult Health. *Frontiers in Endocrinology*.

[40] Kosmas C. E., Bousvarou M. D., Kostara C. E., Papakonstantinou E. J., Salamou E., Guzman E. (2023). Insulin Resistance and Cardiovascular Disease. *Journal of International Medical Research*.

[41] Che B., Zhong C., Zhang R. (2023). Triglyceride-Glucose Index and Triglyceride to High-Density Lipoprotein Cholesterol Ratio as Potential Cardiovascular Disease Risk Factors: An Analysis of UK Biobank Data. *Cardiovascular Diabetology*.

[42] Hill M. A., Yang Y., Zhang L. (2021). Insulin Resistance, Cardiovascular Stiffening and Cardiovascular Disease. *Metabolism*.

[43] Muniyappa R., Chen H., Montagnani M., Sherman A., Quon M. J. (2020). Endothelial Dysfunction Due to Selective Insulin Resistance in Vascular Endothelium: Insights From Mechanistic Modeling. *American Journal of Physiology-Endocrinology and Metabolism*.

[44] Babiker A., Hassan M., Muhammed S. (2020). Inflammatory and Cardiovascular Diseases Biomarkers in Chronic Hepatitis C Virus Infection: A Review. *Clinical Cardiology*.

[45] Sullivan S., Hammadah M., Wilmot K. (2018). Young Women With Coronary Artery Disease Exhibit Higher Concentrations of Interleukin-6 at Baseline and in Response to Mental Stress. *Journal of the American Heart Association*.

[46] Vajdi M., Ardekani A. M., Nikniaz Z., Hosseini B., Farhangi M. A. (2023). Dietary Insulin Index and Load and Cardiometabolic Risk Factors Among People With Obesity: A Cross-Sectional Study. *BMC Endocrine Disorders*.

[47] Zhang Y., Du M., Li Z. The Correlation Between Visceral Fat Area to Skeletal Muscle Mass Ratio and Multi-Organ Insulin Resistance in Chinese Population with Obesity: A Cross-Sectional Study. *05 September 2023, PREPRINT (Version 1) available at Research Square*.

